# Severe mental illness: cardiovascular risk assessment and management

**DOI:** 10.1093/eurheartj/ehae054

**Published:** 2024-02-21

**Authors:** Christoffer Polcwiartek, Kevin O’Gallagher, Daniel J Friedman, Christoph U Correll, Marco Solmi, Svend Eggert Jensen, René Ernst Nielsen

**Affiliations:** Department of Cardiology, Aalborg University Hospital, Hobrovej 18-22, DK-9000 Aalborg, Denmark; Cardiovascular Department, King’s College Hospital NHS Foundation Trust London, London, UK; School of Cardiovascular and Metabolic Medicine & Sciences, British Heart Foundation Centre of Research Excellence, King’s College London, London, UK; Section of Cardiac Electrophysiology, Duke University Medical Center, Durham, NC, USA; Department of Psychiatry, The Zucker Hillside Hospital, Northwell Health, Glen Oaks, NY, USA; Department of Psychiatry and Molecular Medicine, Donald and Barbara Zucker School of Medicine at Hofstra/Northwell, Hempstead, NY, USA; Center for Psychiatric Neuroscience, The Feinstein Institute for Medical Research, Northwell Health, New Hyde Park, NY, USA; Department of Child and Adolescent Psychiatry, Charité Universitätsmedizin Berlin, Berlin, Germany; DZPG, German Center for Mental Health, Partner Site Berlin, Berlin, Germany; Department of Child and Adolescent Psychiatry, Charité Universitätsmedizin Berlin, Berlin, Germany; Department of Psychiatry, University of Ottawa, Ontario, Canada; Regional Centre for the Treatment of Eating Disorders and On Track: The Champlain First Episode Psychosis Program, Department of Mental Health, The Ottawa Hospital, Ontario, Canada; Ottawa Hospital Research Institute (OHRI), Clinical Epidemiology Program, University of Ottawa, Ontario, Canada; Department of Cardiology, Aalborg University Hospital, Hobrovej 18-22, DK-9000 Aalborg, Denmark; Department of Clinical Medicine, Aalborg University, Aalborg, Denmark; Department of Clinical Medicine, Aalborg University, Aalborg, Denmark; Psychiatry, Aalborg University Hospital, Aalborg, Denmark

**Keywords:** Severe mental illness, Cardiovascular disease, Risk factors, Screening, Cardiovascular mortality, Sudden cardiac death, Risk stratification

## Abstract

Patients with severe mental illness (SMI) including schizophrenia and bipolar disorder die on average 15–20 years earlier than the general population often due to sudden death that, in most cases, is caused by cardiovascular disease. This state-of-the-art review aims to address the complex association between SMI and cardiovascular risk, explore disparities in cardiovascular care pathways, describe how to adequately predict cardiovascular outcomes, and propose targeted interventions to improve cardiovascular health in patients with SMI. These patients have an adverse cardiovascular risk factor profile due to an interplay between biological factors such as chronic inflammation, patient factors such as excessive smoking, and healthcare system factors such as stigma and discrimination. Several disparities in cardiovascular care pathways have been demonstrated in patients with SMI, resulting in a 47% lower likelihood of undergoing invasive coronary procedures and substantially lower rates of prescribed standard secondary prevention medications compared with the general population. Although early cardiovascular risk prediction is important, conventional risk prediction models do not accurately predict long-term cardiovascular outcomes as cardiovascular disease and mortality are only partly driven by traditional risk factors in this patient group. As such, SMI-specific risk prediction models and clinical tools such as the electrocardiogram and echocardiogram are necessary when assessing and managing cardiovascular risk associated with SMI. In conclusion, there is a necessity for differentiated cardiovascular care in patients with SMI. By addressing factors involved in the excess cardiovascular risk, reconsidering risk stratification approaches, and implementing multidisciplinary care models, clinicians can take steps towards improving cardiovascular health and long-term outcomes in patients with SMI.

## Graphical Abstract

**Graphical Abstract:**
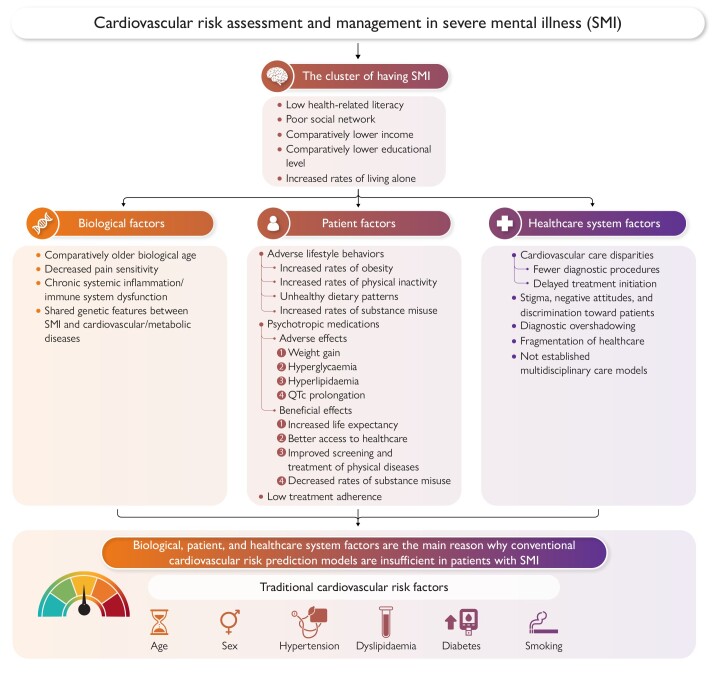
The increased risk of cardiovascular disease associated with severe mental illness (SMI) is multifactorial and driven by complex pathways. Patients with SMI have a high burden of cardiovascular risk factors and disease, and a major challenge in clinical practice is that these patients tend to neglect cardiovascular symptoms, healthcare professionals often overlook and misinterpret cardiovascular signs and symptoms of patients, patients are less likely to receive timely and proper cardiovascular care, and treatment adherence is reduced in these patients. Consequently, patients with SMI develop significantly earlier atherosclerotic cardiovascular disease and therefore require intensive primary prevention strategies that include a more aggressive approach to management of cholesterol levels, blood pressure, and other cardiovascular risk factors.

## Introduction

Approximately 20% of individuals in Europe suffer from mental illness, and the lifetime prevalence of schizophrenia and bipolar disorder, often jointly termed as severe mental illness (SMI), is nearly 2%.^1^ Although major depression clinically also fulfil criteria for SMI and is associated with adverse cardiovascular outcomes, we do not focus on major depression in this state-of-the-art review because it has been extensively studied regarding cardiovascular disease^2^ and is recognized as an independent cardiovascular risk factor in several contemporary international cardiovascular treatment guidelines.^3–5^

Schizophrenia and bipolar disorder generally have a chronic and episodic clinical course, with intermittent psychotic symptoms in most patients with schizophrenia^6^ and with intermittent periods of mood and energy fluctuations where psychotic features are less pronounced in patients with bipolar disorder.^7^ Overall, SMI adversely impacts cognitive, behavioural, and emotional domains of patients, which affects functioning in daily life activities.^8^

A major public health concern is that patients with SMI have a reduced life expectancy of 15–20 years relative to the general population, with an increased risk of sudden death. The leading cause of early death in the general population is cardiovascular disease. This is also true in patients with SMI. However, the risk of cardiovascular mortality and sudden cardiac death (SCD) is up to five-fold higher in patients with SMI,^9^ across both sexes, all ages, and all ethnic groups.^10^ Both the incidence of cardiovascular disease and mortality have declined in the general population over recent decades, primarily due to adjustments in lifestyle behaviour, advances in cardiac interventions, and increased use of guideline-directed medical therapy.^11^ However, patients with SMI have not benefitted similarly from these interventions on total cardiovascular risk reduction^12^; thus, a mortality gap persists that even may be widening.^13–16^

Cardiovascular mortality rates in patients with SMI have been found to be increased based on mainly registry-based studies,^16–18^ but this approach carries a risk of misclassifying specific causes of death. Interestingly, autopsy studies in patients with SMI have not clearly demonstrated an increase in coronary artery calcification (CAC).^19^ The use of CAC scores obtained from cardiac computed tomography (CT) scans does not appear to be of additional predictive value in patients with SMI.^20,21^ Data suggest that, even in patients with a low CAC score, there is still increased mortality in patients with SMI that is three- to four-fold that of the non-SMI population.^20^ At the time of CAC scoring, patients with SMI tend to be younger with higher proportions of smoking, hypertension, overweight, and diabetes.^20^ Furthermore, follow-up studies after myocardial infarction reveal a mortality rate similar to that observed in SMI but not in myocardial infarction compared with the general population.^15^ Overall, these findings suggest that while cardiovascular mortality rates may be increased in patients with SMI, the underlying mechanisms and specific cardiovascular risk factors require further investigation to better understand the association with SMI.

In this state-of-the-art review, we aimed to address the complex relationship between SMI and cardiovascular risk factors, disease, and mortality as well as disparities in cardiovascular care pathways. We further described how to adequately predict future cardiovascular disease events in the setting of SMI. Finally, we proposed strategies and future directions regarding targeted interventions to improve cardiovascular health in patients with SMI.

## Cardiovascular risk in severe mental illness

The excess cardiovascular disease burden associated with SMI is attributable to factors that can be addressed from a biological, patient, and healthcare system perspective. These factors represent potential barriers and challenges that can be specifically targeted, either alone or in combination, to improve cardiovascular health and long-term outcomes in patients with SMI, as shown in the *Graphical Abstract*.

### Biological factors

The American Heart Association has recognized that SMI predisposes young patients to an early onset of atherosclerotic cardiovascular disease (ASCVD) and consequently accelerated incidence of myocardial infarction and heart failure, with an associated increase in mortality over time.^22^ Emerging evidence supports that SMI has been associated with an older functional biological age, resulting in earlier onset of cardiovascular risk factors and disease.^23,24^ This fact underscores the importance of strict management of cardiovascular risk factors, early cardiovascular screening, and more intensive cardiovascular treatment in patients with SMI relative to the general population.^9^

Abnormal pain threshold or decreased pain sensitivity^25^ has been suggested to be an important mechanism to the increased risk of unrecognized cardiovascular disease in patients with SMI and may explain why a subset of patients present with more advanced stages of physical disease. Of note, the rate of myocardial infarction that is unrecognized, but identified by pathological Q-waves on the electrocardiogram (ECG) as a sign of myocardial scarring, in patients with SMI has been reported to be as high as 75%–85%.^26,27^ However, such studies would benefit from confirmatory work in the era of cardiac magnetic resonance imaging (MRI). Although the mechanism is poorly understood, it has been suggested that the abnormal pain threshold or decreased pain sensitivity is more related to a different mode of pain expression than to a real endogenous analgesia.^28^

Research suggests that subclinical chronic systemic inflammation and dysfunction of the immune system are involved in schizophrenia and other chronic neuropsychiatric disorders.^29^ Although acute inflammation represents an adaptive body response, the long-term consequences of persistent systemic inflammation and activation of the immune system may be harmful in that it has been associated with the development of obesity, insulin resistance, type 2 diabetes, acute coronary syndrome (ACS), and metabolic syndrome.^30,31^

Recent data also support shared genetic features between SMI and cardiovascular and metabolic diseases, considering that altered cardiac structure and function have been observed in patients with SMI free of cardiovascular risk factors and disease as well as after adjustment for both lifestyle and medication effects. Polygenic risk score analyses have demonstrated that particularly patients with schizophrenia may be genetically predisposed to cardiometabolic disease, whereas this genetic predisposition is not observed in patients with bipolar disorder.^32^ Novel evidence also supports that cardiac MRI findings including several left ventricular parameters have been genetically correlated with SMI.^33^

### Patient factors

In patients with SMI, there is an associated two- to four-fold higher rate of diabetes, hypertension, dyslipidaemia, and metabolic syndrome compared with the general population, as shown in *Table 1*. Furthermore, these patients are more likely to smoke, be obese, have obstructive sleep apnoea, live a sedentary lifestyle, and eat unhealthy.^34,35^ Most of the traditional cardiovascular risk factors are at least to some extent present from the first episode of SMI^36^ and correlate with illness duration,^37^ with a more rapid worsening than in patients without SMI.^38^ Although there is a systematic underrecognition and undertreatment of both cardiovascular risk factors and disease in patients with SMI, these patients have higher rates of hospitalizations or outpatient visits when subsequently diagnosed with a cardiovascular disease compared with the general population.^39^

**Table 1:** Prevalence and relative risk estimates of traditional cardiovascular risk factors in patients with SMI compared with the general population^34^

Risk factor	Schizophrenia	Bipolar disorder
Obesity	45%–55%, RR: 1.5–2	21%–49%, RR: 1–2
Smoking	50%–80%, RR: 2–3	54%–68%, RR: 2–3
Diabetes	10%–15%, RR: 2	8%–17%, RR: 1.5–2
Hypertension	19%–58%, RR: 2–3	35%–61%, RR: 2–3
Dyslipidaemia	25%–69%, RR: ≤ 5	23%–38%, RR: ≤ 3
Metabolic syndrome	37%–63%, RR: 2–3	30%–49%, RR: 1.5–2

RR, relative risk; SMI, severe mental illness.

In particular, smoking is highly prevalent in patients with SMI, with some studies reporting rates up to 80%.^40^ Smoking cessation may be one of the most effective measures for total cardiovascular risk reduction in patients with SMI, and the need for effective smoking cessation interventions is underscored by epidemiological data demonstrating that patients with schizophrenia who smoke have an 86% higher 20-year cardiovascular mortality risk than non-smoking patients with schizophrenia.^41^ Smoking cessation interventions are underutilized in patients with SMI but are generally as effective as in the general population, with specialized treatment (e.g. bupropion and varenicline) showing improved long-term efficacy and high safety.^42,43^

There is also a complex interaction between markers of metabolic syndrome, SMI, and antipsychotic treatment. Although antipsychotics have adverse effects on cardiovascular risk by promoting weight gain, hyperglycaemia, and hyperlipidaemia, they have overall net benefits on all-cause and specifically cardiovascular mortality.^16^ This seeming paradox^44^ is likely explained by greater psychiatric stability through antipsychotic treatment translating into improved healthy lifestyle behaviours, reduced psychotic-related stress, and greater adherence to medical interventions, thus reducing cardiometabolic risk factors and disease,^45^ which may potentially be a proxy for better access to healthcare when on treatment.

Due to the nature of SMI, adherence to medication is a significant challenge, and patients are often suffering from lack of awareness of physical symptoms, cognitive impairment, social deprivation, and poor self-care.^9^ In particular, social deprivation has been associated with poor quality of care and outcomes in patients with ischaemic heart disease as well as associated with poor control of cholesterol and blood sugar levels.^46,47^ In addition, cardiovascular risk factors can also worsen cognitive functioning,^48^ further contributing to non-adherence and poor functioning in patients with SMI.

### Healthcare system factors and disparities in cardiovascular care pathways

The intersection of SMI and cardiovascular health poses significant challenges within healthcare systems.^35^ Patients with SMI often face disparities in accessing and receiving appropriate cardiovascular care, and a main factor influencing this disparity is the pervasive stigma and discrimination surrounding mental illness.^49,50^ These negative attitudes can lead to healthcare providers overlooking or downplaying physical symptoms in patients with SMI. Similarly, patients with SMI may have mental symptoms overshadowing the co-occurring physical disease (e.g. diagnostic overshadowing).^51^ Consequently, cardiovascular risk factors may be neglected or inadequately addressed, increasing the likelihood of adverse cardiovascular outcomes. A prior study has found that the likelihood of undergoing cardiac interventions including coronary revascularization, arrhythmia treatment, and valve and vascular surgery prior to cardiovascular mortality was lower in patients with SMI compared with the general population. In contrast, the rate of diagnostic tests in primary care settings including ECG testing, cholesterol and diabetes screening, and blood pressure monitoring was similar.^52^

Fragmentation of care across different healthcare providers and specialties is another important factor contributing to disparities in cardiovascular care pathways. Patients with SMI often receive treatment from multiple providers including mental health specialists, primary care physicians, and cardiologists. Communication gaps and lack of care coordination between these providers can result in suboptimal management of cardiovascular risk factors and inadequate integration of mental and physical healthcare.^53^

Among healthcare professionals, potential insufficient clinical training and awareness about the complex relationship between SMI and cardiovascular health can impede effective care delivery. Mental health specialists may not possess comprehensive knowledge of cardiovascular risk assessment and management, while cardiovascular specialists may have limited understanding of the unique challenges faced by patients with SMI. Of note, screening for cardiovascular risk factors in patients with SMI occurs at a lower level than that recommended in guidelines at psychiatric and primary care settings.^54–56^ As such, this knowledge gap may block the development of tailored and multidisciplinary care approaches for this patient population.

## Cardiovascular disease in severe mental illness

### Ischaemic heart disease, revascularization, and coronary artery calcification

In the most extensive meta-analysis to date that included 92 studies and compared epidemiological data from >3 million patients with SMI and >110 million general population controls, SMI was associated with a 54% increased long-term risk of ischaemic heart disease including myocardial infarction.^17^ Furthermore, several retrospective studies have demonstrated that patients with SMI presenting with myocardial infarction are younger with higher rates of cardiovascular risk factors such as smoking and diabetes.^57^ In keeping with the abovementioned discussion of factors precipitating acute cardiovascular disease events in SMI, patients are more likely to have atypical symptoms of myocardial infarction such as atypical angina or dyspnoea^58^ and longer symptom duration^26,27^ that may result in diagnostic delay and prolonged door-to-balloon time, thereby potentially increasing risks of cardiovascular mortality, ischaemic cardiomyopathy and heart failure, as well as ventricular arrhythmias.

An important starting point for any discussion regarding coronary revascularization in patients with SMI is that although there are particular considerations to be made, there is no procedural barrier to performing revascularization procedures in this patient population, meaning it is not more procedurally difficult to perform percutaneous coronary intervention or coronary artery bypass grafting. Overall, considerations can be grouped into pre-procedural issues (i.e. capacity to consent and appropriateness for invasive strategy) and post-procedural issues (i.e. risk of delirium, deterioration of SMI symptoms, and, most importantly, compliance to antiplatelet treatment). However, patients with SMI are less likely to undergo coronary revascularization procedures,^12^ a finding that persists even for those who underwent invasive diagnostic coronary angiography.^57^ Altogether, patients with SMI have a 47% lower likelihood of undergoing cardiac interventions.^59^ Compared with controls, following an ACS event, patients with SMI have increased in-hospital mortality, and long-term post-ACS survival is worse in patients with SMI compared with the general population.^57^ Of note, consistent findings have been observed in patients with SMI presenting with out-of-hospital cardiac arrest, where these patients are less likely to have initial shockable rhythm and a lower likelihood of undergoing cardiac interventions.^60^

A validated clinical marker for predicting ischaemic heart disease events in asymptomatic individuals in the general population remains the Agatston score, a measure of CAC derived from cardiac CT scans.^5^ However, the association between CAC burden and adverse cardiovascular outcomes has not been consistently observed in patients with SMI,^20^ and a recent study in patients with schizophrenia further found similar CAC scores to that of the general population.^21^ It is possible that prior studies only included patients who had a clinical indication for CAC score testing, thereby excluding asymptomatic patients with SMI with potentially high CAC scores. In addition, it can be speculated that patients with SMI with a CAC score of 0 have not yet developed detectable calcified coronary plaques, but they may have fatty streaking and early stages of soft and fibrous plaques that pose a high risk for myocardial infarction, although studies are warranted to support this hypothesis in patients with SMI. Nonetheless, an autopsy study in patients with SMI demonstrated an association between spectroscopy-imaged calcium quantification and pathological calcification on macroscopic and histological examination, thus supporting the clinical utility of CAC score testing in this patient population.^19^

### Ventricular arrhythmias and sudden cardiac death

Up to 66% of patients with SMI have undetected cardiovascular disease prior to cardiovascular death,^61^ and a frequent manifestation of undetected cardiovascular disease may be ventricular arrhythmias (i.e. ventricular tachycardia and fibrillation) and SCD, with the latter being up to five-fold more common in patients with SMI than in the general population.^62^ This represents significant clinical challenges in patients with SMI, but the exact mechanisms behind the association are not fully understood. However, the risk appears to be more pronounced in younger age groups and is independent of cardiovascular risk factors.^62^

The pro-arrhythmic effect of psychotropic medications including antipsychotics and antidepressants is an important factor in understanding the increased arrhythmogenic risk in patients with SMI.^63^ Antipsychotic treatment has been associated with various degrees of risk of corrected QT (QTc) interval prolongation on the ECG,^64^ ventricular arrhythmias, particularly the polymorphic ventricular tachycardia torsades de pointes, and SCD. In patients exposed to antipsychotics, SCD occurs twice as often compared with non-exposed patients, with an incidence of ∼15/10 000 years of exposure.^65,66^ In a prior study, antipsychotic exposure has also been strongly associated with an increased risk of out-of-hospital cardiac arrest, although it was unclear whether the cardiac arrest was preceded by an arrhythmic event.^67^

Other potential factors associated with ventricular arrhythmias and SCD in patients with SMI involve autonomic dysfunction, chronic inflammation, oxidative stress, and genetic predisposition. In particular, autonomic dysfunction, characterized by increased sympathetic tone and reduced parasympathetic activity, is common in patients with SMI^68,69^ and may alter cardiac repolarization, reduce heart rate variability, and cause ventricular arrhythmias.^70^ Chronic inflammation and oxidative stress have also been associated with pro-arrhythmic effects by altering ion channel function and calcium handling in cardiomyocytes, leading to increased arrhythmogenicity.^71^

### Heart failure and altered cardiac structure and function

Heart failure in patients with SMI is a concerning cardiovascular complication secondary to the increased cardiovascular risk and occurs often at an earlier age than in the general population.^72^ Of note, SMI has been associated with a more than two-fold increased long-term risk of heart failure compared with the general population.^17^ It is well known that cardiovascular risk factors such as hypertension and diabetes as well as cardiovascular disease such as myocardial infarction and atrial fibrillation, which often are underdiagnosed and undertreated in patients with SMI, play a pivotal role in the development of heart failure.^4^ However, chronic inflammation, oxidative stress, and neurohormonal imbalance are also important factors in the pathophysiology of heart failure, contributing specifically to left ventricular remodelling and dysfunction.^73^ Furthermore, neurohormonal imbalance has been associated with increased sympathetic nervous system activity and altered renin–angiotensin–aldosterone system regulation as a compensatory mechanism, which is responsible for increased preload and afterload in early stages of heart failure.^70^

In addition to the increased rates of cardiovascular risk factors and disease, antipsychotic exposure has been associated with reduced left ventricular ejection fraction in patients with schizophrenia, and the risk is especially pronounced in those treated with clozapine.^74–76^ Furthermore, high polygenic risk scores for schizophrenia have recently been associated with myocardial stiffness and decreased absolute peak diastolic strain rates (i.e. diastolic dysfunction), which may increase the risk of heart failure.^77^ Overall, the decreased left ventricular ejection fraction and diastolic dysfunction may be a direct result of the antipsychotic treatment or, in combination with the SMI, associated with early diffuse myocardial fibrosis and subclinical myocardial inflammation, contributing to the development of left ventricular concentric remodelling.^78^ In contrast, patients with bipolar disorder on lithium treatment have not been found to experience altered left ventricular systolic and diastolic function compared with the general population.^79^

The complex association between heart failure, left ventricular dysfunction, and SMI has only been scarcely investigated. It appears that long-term heart failure outcomes in patients with SMI are adverse across the whole heart failure spectrum (i.e. heart failure with reduced, mid-range, and preserved ejection fraction) and worst for males. The rates for advanced heart failure treatment including implantable cardioverter defibrillator use, cardiac resynchronization therapy, left ventricular assist device implantation, and heart transplantation are similar between patients with SMI and the general population, but patients with SMI undergoing these procedures have an increased all-cause mortality risk.^72^ Overall, early evaluation and recognition of concomitant heart failure and SMI are important to reduce potential disparities in heart failure care pathways. However, patients with SMI hospitalized for heart failure tend to receive fewer echocardiograms for left ventricular ejection fraction evaluation, despite higher rates of heart failure rehospitalizations and 1-year all-cause mortality.^80^

### Atrial fibrillation and oral anticoagulation treatment

Atrial fibrillation is the most prevalent arrhythmia and associated with heart failure and a five-fold increased risk of stroke, leading to death in some cases.^81^ Although patients with SMI have a 78% higher risk of developing cardiovascular disease compared with the general population,^17^ the rate of atrial fibrillation has been reported to be lower in patients with SMI than in the general population after controlling for age and sex.^82,83^ Importantly, this finding may also reflect potential underdiagnosis of atrial fibrillation in this patient population, and diagnostic overshadowing may play an important factor as symptoms such as palpitations or dyspnoea may be misinterpreted by clinicians or patients.

3Despite high-quality evidence of a substantial net benefit from oral anticoagulation treatment in general population controls with atrial fibrillation, patients with SMI and atrial fibrillation have been found to be less likely to receive oral anticoagulants and adhere to treatment, with risks of stroke and major bleeding being increased in this patient population.^83^

### Myocarditis

Certain antipsychotics have been associated with myocarditis due to a type 1 hypersensitivity reaction with eosinophilic predominance, where abundant eosinophils may release toxins and induce apoptosis and necrosis of cardiomyocytes.^66^ Clozapine is by far the antipsychotic that has been associated with myocarditis the most, although the rate is generally low (varying from <0.1% in some countries to as high as 3% in Australia).^84^ Risk factors for antipsychotic-associated myocarditis include recent viral, bacterial, or parasitic infections, a history of myocardial infarction, pericarditis, cardiomyopathy, or heart failure,^66^ and in relationship to clozapine, a confirmed ‘allergic reaction’ that is possibly mostly related to too rapid clozapine titration that induces inflammation.^85^

Myocarditis may mimic ACS in patients with SMI prescribed antipsychotics and result in myocardial fibrosis, ventricular arrhythmias, and potentially SCD. Myocarditis generally occurs within the first months of antipsychotic treatment initiation and is diagnosed by potential ST-segment deviations on the ECG and increased cardiac troponin levels. Patients may present with flu-like symptoms, fever, fatigue, angina, or dyspnoea.^66^ Potential cases of antipsychotic-associated myocarditis should always be referred for specialist cardiology assessment.

### Cardiac surgery and other cardiac conditions

Patients with SMI have been found to have a higher rate of mitral and aortic valve disease than the general population.^86,87^ Evidence for cardiac surgery remains largely scarce, but few case reports have reported favourable outcomes in patients with SMI undergoing valve replacement.^88^

Right-sided infective endocarditis is common in intravenous drug abusers,^89^ and patients with SMI are specifically susceptible because of the high rate of intravenous drug abuse in this population.^90^

Long-term epidemiological data on bradyarrhythmias, pacemaker implantation procedures, and related outcomes in patients with SMI are lacking, and only few cases have been reported in the literature.

## Cardiovascular preventive strategies in severe mental illness

### Primary prevention and cardiovascular risk prediction models

Based on the abovementioned biological, patient, and healthcare system factors that are involved in the excess cardiovascular risk in patients with SMI, tailored primary prevention strategies are critical to reduce the burden of cardiovascular disease, improve long-term outcomes, and reduce the mortality gap.^35^ Regular cardiovascular risk assessment including blood pressure monitoring and evaluation of metabolic parameters is important to screen for and detect cardiovascular risk factors early in this patient population.^91^ However, screening and monitoring of cardiovascular risk factors in patients with SMI are generally suboptimal.^92,93^ Concerningly, the rate of patients with SMI not treated for established cardiovascular risk factors has been reported to be as high as 88% for dyslipidaemia, 62% for hypertension, and 30% for diabetes.^94–96^

The primary contributor to the total attributable risk for ASCVD is the lipid profile, particularly elevated LDL cholesterol (LDL-C) levels.^97,98^ In addition, lipoprotein(a) [Lp(a)] elevations are very common, as an almost pure genetic factor increasing risks of ASCVD and calcific aortic valve stenosis. According to a recent consensus statement, Lp(a) should serve as a risk stratifier and be measured at some point in all adults undergoing lipid profile testing, preferably in their initial assessment.^99^ The inclusion of Lp(a) in the overall risk assessment improves ASCVD risk stratification, especially for patients with extremely high Lp(a) levels. In one study, 31%–63% of those with Lp(a) levels >99th percentile were reclassified from moderate to higher risk.^100^ Considering these observations, a more aggressive approach to lipid management is likely to provide the greatest benefit in reducing ASCVD risk for patients with SMI. The 2019 ESC/EAS Guidelines for the management of dyslipidaemias^101^ recognize SMI as a significant risk enhancer, suggesting to employ SMI as a modifier when assessing overall ASCVD risk. However, the guidelines for managing total ASCVD risk in patients with SMI align with those applied to patients without SMI. Although statin treatment is equally effective in lowering LDL-C levels in patients with SMI as in the general population, statins are underutilized in these patients, despite their substantially higher mortality risk from ASCVD.^101^ Future studies should investigate specific targets for LDL-C lowering in combination with Lp(a) levels and their association with long-term ASCVD outcomes in patients with SMI, aiming to assist clinicians in timely risk stratifying patients into low, moderate, or high risk.

Promoting lifestyle interventions including physical activity, healthy eating habits, smoking cessation, and drug abuse treatment is essential to reduce the cardiovascular risk in this patient population.^102,103^ However, the positive effects of such non-pharmacological lifestyle interventions, if observed, have not proven very effective and are generally not cost effective in patients with SMI.^104–106^ In addition, mental health specialists should carefully assess cardiovascular adverse effects of psychotropic medications and consider alternative agents with a more favourable cardiometabolic profile when treating patients with SMI.^66,107^

Early cardiovascular risk prediction is crucial to determine the need for initiation of preventive measures as many patients with SMI may experience SCD as the initial manifestation of undetected cardiovascular disease. Various prediction models including the SCORE (Systematic COronary Risk Evaluation)^5^ and the Framingham Risk Score^108^ have been proposed to estimate the 10-year risk of cardiovascular disease events. However, these models have been found to underestimate cardiovascular risk in patients with SMI as traditional risk factors only partially explain the excess cardiovascular mortality observed in this patient population.^109^ As a result, the PRIMROSE (PRedIction and Management of cardiovascular Risk in peOple with SEvere mental illnesses) lipid model and the PRIMROSE body mass index model have been specifically developed and validated to predict the 10-year risk of incident cardiovascular disease events in patients with SMI.^110^ The PRIMROSE model demonstrated that additional risk factors contributed to the development of cardiovascular disease including social deprivation, SMI subtype, prescriptions for antidepressants and antipsychotics, and reports of alcohol abuse. In patients with SMI, the PRIMROSE model predicted incident fatal and non-fatal cardiovascular disease events much more strongly than the comparator Framingham Risk Score model.^110^ The PRIMROSE intervention study examined a nurse-led intervention for reducing cardiovascular risk, using a cluster randomization of general practices, that included adults with SMI, a total cholesterol of >5.0 mmol/L, and an additional cardiovascular risk factor. This intervention had no beneficial effects on cholesterol levels or statin use, the use of which remained low. This cluster randomized trial tested a programme with no specific targets for lipid management or blood pressure control.^111^

The standard 12-lead ECG remains one of the most important clinical tools in the diagnosis and risk stratification of cardiovascular disease in patients with SMI, and it has been demonstrated that adding ECG abnormalities to a conventional cardiovascular risk prediction model increased the area under the curve for the 10-year absolute risk prediction of cardiovascular mortality in patients with SMI compared with the general population. Furthermore, both minor and major ECG abnormalities conferred increased long-term cardiovascular mortality compared with general population controls, suggesting high clinical utility of the ECG regarding cardiovascular risk assessment in the SMI population.^112^

### Secondary prevention of cardiovascular disease

Addressing secondary prevention in patients with SMI is crucial due to their increased risk of cardiovascular disease and high rate of associated risk factors. It is reasonable that these patients may benefit more from the standard secondary prevention medications that are offered to the general population following a diagnosis of cardiovascular disease. However, several barriers are present as to why secondary prevention strategies are difficult to implement in this patient population. Most importantly, poor medication adherence is common in patients with SMI, leading to suboptimal cardiovascular care.^9^

Lower prescription rates of cardiovascular medications have overall been observed in patients with SMI compared with the general population.^12,113^ The most extensively researched treatment gap has been performed for post-ACS care, where patients with SMI following myocardial infarction were less likely to receive aspirin, P2Y_12_ inhibitors, beta-blockers, statins, angiotensin-converting enzyme inhibitors or angiotensin II receptor blockers, or mineralocorticoid receptor antagonists.^58,114–116^ A registry-based study, investigating the association between exposure to standard secondary prevention medications following myocardial infarction and all-cause mortality risk, demonstrated that patients with schizophrenia experienced a decrease in mortality risk from a hazard ratio of 8.78 to 1.97 with secondary prevention treatment. In contrast, in the general population, the absence of secondary prevention treatment increased the mortality risk from a hazard ratio of 1.00 (reference) to 2.95.^117^

## Perspectives on improving cardiovascular health in patients with severe mental illness

A box table summarizing key clinical recommendations regarding cardiovascular risk assessment and management in patients with SMI is shown in *Table 2*.

**Table 2:** Box table summarizing key clinical recommendations regarding cardiovascular risk assessment and management in patients with SMI

**1. Overall goals**
(a) To establish equality in the management of cardiovascular risk factors and treatment of cardiovascular disease and ensure sufficient accessibility to cardiovascular examinations for patients with SMI, comparable with the standards observed in the general population.
(b) To adhere to contemporary international cardiovascular treatment guidelines in the management of patients with SMI.
**2. Primary prevention strategy goals**
(a) Hypertension is frequent in patients with SMI, but antihypertensive treatment is often underutilized or patients are undertreated. Patients should be screened for hypertension early and regularly. Blood pressure control and timely initiation of sufficient antihypertensive treatment are essential to mitigate cardiovascular disease risk in these patients.
(b) Dyslipidaemia plays an important role in the premature development of atherosclerotic cardiovascular disease in patients with SMI. Patients, even without other risk-enhancing factors, may potentially benefit from a more aggressive approach to lipid management. Although statin treatment is equally effective in lowering LDL-C levels in patients with SMI as in the general population, statins are underutilized in these patients.
(c) Diabetes is common at an early stage in patients with SMI, often exacerbated by antipsychotic treatment by altering glucose metabolism and promoting weight gain. Regular monitoring of HbA1c levels to assess long-term glucose control is important. Treatment plans should be tailored to both individual needs and comorbidities including ischaemic heart disease, heart failure, or nephropathy, where SGLT2 inhibitors or GLP-1 RAs are indicated. Management should extend beyond medications to include lifestyle modifications and collaborative care.
(d) Smoking rates are increased in patients with SMI, and smoking cessation interventions are generally underutilized. Specialized treatment such as bupropion and varenicline shows improved long-term efficacy and high safety in these patients.
(e) Physical inactivity, obesity, unhealthy diet, and substance misuse in patients with SMI are also highly prevalent cardiovascular risk factors. Clinicians are strongly encouraged to actively screen for these risk-enhancing factors and implement targeted management strategies by providing guidance on lifestyle interventions, patient education, and integrated care approaches.
**3. Secondary prevention strategy goals**
(a) Patients with SMI may potentially benefit more from the standard secondary prevention treatment that is offered to the general population following a diagnosis of cardiovascular disease.
(b) The largest treatment gap is potentially within post-ACS care, where patients with SMI and myocardial infarction are less likely to receive aspirin, P2Y_12_ inhibitors, beta-blockers, statins, ACEIs/ARBs, or MRAs compared with the general population. The majority of patients with SMI have a lower likelihood of undergoing invasive coronary procedures. When treated sufficiently, no differences in post-myocardial infarction mortality are observed between patients with SMI and the general population.
**4. Integrated healthcare goals**
(a) Multidisciplinary care models bridging the gap between mental health, primary care, and cardiology can contribute to more comprehensive and effective management of the excess cardiovascular risk in patients with SMI. A comprehensive and individualized approach to managing risk factors is essential for optimizing cardiovascular health in the SMI population.

ACEIs, angiotensin-converting enzyme inhibitors; ARBs, angiotensin receptor blockers; ACS, acute coronary syndrome; GLP-1 RAs, glucagon-like peptide-1 receptor agonists; HbA1c, glycated haemoglobin; LDL-C, LDL cholesterol; MRAs, mineralocorticoid receptor antagonists; SGLT2, sodium–glucose cotransporter 2; SMI, severe mental illness.

Multidisciplinary care models combining mental health and cardiovascular care are essential in addressing the unique challenges faced by patients with SMI and improving their overall cardiovascular health and long-term outcomes.^9,118^ Primary prevention strategies play a crucial role in mitigating cardiovascular risk and improving long-term outcomes, while tailored approaches including medication management, lifestyle interventions, and psychosocial support can enhance secondary prevention strategies in patients with SMI. Overall, collaborative efforts between mental health specialists, cardiologists, primary care physicians, and other healthcare professionals are important for effective primary and secondary prevention in this vulnerable patient population.^35^ To achieve improved long-term cardiovascular outcomes and reduce the excess cardiovascular risk in patients with SMI, further research is warranted to understand the complex relationship between mental health and cardiovascular disease events, ideally paving the way towards more targeted interventions.

Implementing multidisciplinary care models that promote collaboration across medical specialties can enhance cardiovascular care pathways for patients with SMI. For example, in mental health and primary care settings, ECGs are routinely obtained for assessing QTc interval prolongation while on antipsychotic treatment, and in case the QTc interval is within normal limits but other major ECG abnormalities are present such as evidence of prior myocardial infarction by pathological Q-waves, patients with SMI are not necessarily referred to cardiologists for secondary evaluation, treatment, and prevention. As such, regular communication channels, shared care plans, and joint decision-making processes are essential features of this approach. To optimize patient outcomes, healthcare professionals should receive comprehensive training on the interface between mental health and cardiovascular care. Further research and awareness initiatives are warranted to emphasize the importance of both primary and secondary prevention in patients with SMI and to develop a comprehensive approach to cardiovascular health.

## Conclusions

Much is known about total cardiovascular risk reduction in the general population, and over decades, substantial efforts have been made to improve cardiovascular risk assessment, management, and treatment of cardiovascular disease, especially in the era of myocardial reperfusion and cardiac rehabilitation. Although significant progress has been made to understand the contribution of SMI in cardiovascular disease, patients with SMI have not benefitted from the clinical advances in cardiology compared with the general population. This current review underscores the necessity for personalized cardiovascular care and management of cardiovascular disease in patients with SMI. By addressing factors involved in the excess cardiovascular risk, reconsidering risk stratification approaches, and implementing multidisciplinary care models, clinicians can take steps towards the necessary improvement of cardiovascular health and long-term outcomes in patients with SMI.

## Supplementary data

Supplementary data are not available at *European Heart Journal* online.

## Declarations

### Disclosure of Interest

C.P. has received speaking fees from Lundbeck and research grant support from the Danish Cardiovascular Academy (grant number: CPD5Y-2021003-DCA), funded by the Novo Nordisk Foundation (grant number: NNF20SA0067242) and the Danish Heart Foundation. D.J.F. has received research grants from Abbott, American Heart Association, Biosense Webster, Boston Scientific, Medtronic, Merit Medical, National Cardiovascular Data Registry, Phillips, and National Institutes of Health and consulting fees from Abbott, NI Medical, Microport, and Sanofi. C.U.C. has been a consultant for and/or advisor to or has received honoraria from AbbVie, Acadia, Alkermes, Allergan, Angelini, Aristo, Biogen, Boehringer Ingelheim, Cardio Diagnostics, Cerevel, CNX Therapeutics, Compass Pathways, Darnitsa, Denovo, Gedeon Richter, Hikma, Holmusk, IntraCellular Therapies, Jamjoom Pharma, Janssen/J&J, Karuna, LB Pharma, Lundbeck, MedAvante-ProPhase, MedInCell, Merck, Mindpax, Mitsubishi Tanabe Pharma, Mylan, Neurocrine, Neurelis, Newron, Noven, Novo Nordisk, Otsuka, Pharmabrain, PPD Biotech, Recordati, Relmada, Reviva, Rovi, Sage, Seqirus, SK Life Science, Sumitomo Pharma America, Sunovion, Sun Pharma, Supernus, Takeda, Teva, Tolmar, Vertex, and Viatris. He provided expert testimony for Janssen and Otsuka. He served on a Data Safety Monitoring Board for Compass Pathways, Denovo, Lundbeck, Relmada, Reviva, Rovi, Supernus, and Teva. He has received grant support from Janssen and Takeda. He received royalties from UpToDate and is also a stock option holder of Cardio Diagnostics, Kuleon Biosciences, LB Pharma, Mindpax, and Quantic. M.S. has been a consultant for or has received honoraria from AbbVie, Angelini, Lundbeck, and Otsuka. R.E.N. has been an investigator for Compass Pharmaceuticals, Janssen-Cilag, Sage, and Boehringer Ingelheim for clinical trials, has received speaking fees from Lundbeck, Teva Pharmaceuticals, Janssen-Cilag, and Otsuka Pharmaceuticals, and has acted as advisor to Lundbeck and Janssen-Cilag. All other authors declare no conflicts of interest.

## Data Availability

No data were generated or analysed for or in support of this paper.
